# Autochthonous dengue in two Dutch tourists visiting Département Var, southern France, July 2020

**DOI:** 10.2807/1560-7917.ES.2020.25.39.2001670

**Published:** 2020-10-01

**Authors:** Tom D Vermeulen, Johan Reimerink, Chantal Reusken, Sandra Giron, Peter J de Vries

**Affiliations:** 1 Tergooi Hospital, Hilversum, the Netherlands; 2 Centrum voor infectieziektenbestrijding, Rijks Instituut voor Volksgezondheid en Milieu (RIVM), Bilthoven, the Netherlands; 3 Santé publique France (French National Public Health Agency), Marseille, France

**Keywords:** Dengue, Aedes albopictus, travel related disease, France, the Netherlands, autochthonous

## Abstract

We report dengue virus (DENV) infection in two Dutch tourists who visited Département Var, southern France, in July and August 2020. As some autochthonous dengue cases have occurred in Europe in recent years, awareness among physicians and public health experts about possible intermittent presence of DENV in southern Europe is important to minimise delay in diagnosis and treatment. Quick diagnosis can lead to timely action to contain the spread of vector-borne diseases and minimise transmission.

Travel-related diseases may serve as sentinels of transmission of disease in the visited area. Prompt diagnosis and notification of such diseases may assist in the detection and control of disease outbreaks. When we diagnosed dengue in a Dutch tourist who visited southern France, we coordinated joint action between the patient and clinical and public health experts. This led to rapid international notification and consecutive outbreak control efforts by French authorities. A second, related, Dutch patient with a recent fever was retrospectively also diagnosed with dengue. 

## Patient 1

A previously healthy woman in her 20s (Patient 1) spent a 2-week holiday with relatives in La Croix Valmer, France, from 13 to 31 July 2020. In the first week of August, Patient 1 stayed with other friends in another house nearby. On 1 August, she developed fever accompanied by myalgia in her calves and neck, as well as a painful skin (Day 1 of the disease episode). On Day 5 post onset of symptoms (POS), she was nauseous and vomited once. On Day 6 POS, she returned to the Netherlands. A test for severe acute respiratory syndrome coronavirus 2 (SARS-CoV-2), on a nasopharyngeal swab, was negative. On Day 8 POS, the patient noticed an itchy erythematous rash on her hands and lower legs. On Day 11 POS, she consulted her general practitioner who, suspecting petechiae, referred her to our hospital. In addition to the reported signs and symptoms, she mentioned a blurred, colourful spot in the field of vision of her left eye.

During childhood, the patient was vaccinated according to the Dutch national vaccination scheme; she had never received any vaccinations for yellow fever, Japanese encephalitis or tick-borne encephalitis. Interestingly, one of her family members upon our clinical suspicion of dengue virus infection spontaneously acknowledged having seen tiger mosquitoes (*Aedes albopictus)* around their holiday home. Physical examination showed normal vital signs and revealed slight erythematous exanthema on her hands and upper limbs and a confluent petechiae-like exanthema on both legs. The presumptive diagnosis of dengue was made, common laboratory tests including dengue virus (DENV) serology were ordered and she was referred to the ophthalmologist. Fluorescein angiography of the eyes showed an inflammatory foveolitis in her left eye.

Laboratory results on Day 12 POS showed a mild thrombocytopenia and leukocytopenia with plasmocytosis, and moderately elevated serum levels of the liver enzymes (Table 1). On the same serum, comparative IgM and IgG serology (Immunofluorescence Arbovirus Fever Mosaic 1, EuroImmun AG, Lübeck, Germany) against chikungunya virus (CHIKV), DENV and Japanese encephalitis virus (JEV) was performed at the Dutch National Institute for Public Health and the Environment (RIVM) laboratory. High concentrations of DENV-specific IgM and IgG antibodies were detected (Table 2). There was a slight IgG response, without IgM response, against JEV, interpreted as non-specific cross-reactivity. RT-PCR was not done. 

**Table 1:** Laboratory findings, dengue Patient 1, France, July 2020

	Day 11	Day 12	Normal range
ALAT	381	279	<35 U/L
ASAT	281	134	<30 U/L
GGT	81	81	<27 U/L
LD	366	Not performed	<250 U/L
Thrombocytes	103 × 10^9^/L	113 × 10^9^/L	(150–450) × 10^9^ /L
Leukocytes	3.6 × 10^9^/L	3.4 × 10^9^/L	(4.0–11.0) × 10^9 ^/L

ALAT: alanine aminotranferase; ASAT: aspartate aminotransferase; GGT: gamma-glutamyltransferase; LD: lactate dehydrogenase.

**Table 2:** IgG and IgM serum antibody concentrations (reciprocal titres) against dengue, Japanese encephalitis and chikungunya virus, Patients 1 and 2, France, July 2020

	Patient 1	Patient 2
Dengue virus IgG	1:2,048	1:2,048
Dengue virus IgM	1:2,048	1:512
Japanese encephalitis virus IgG	1:32	1>256
Japanese encephalitis virus IgM	Negative	Negative
Chikungunya virus IgG	Negative	Negative
Chikungunya virus IgM	Negative	Negative

## Patient 2

Patient 2 spent the holiday in la Croix Valmer, together with Patient 1, from 13 to 31 July. He returned to the Netherlands 1 week before Patient 1. On 1 August, he noticed a mild pain in his right ear (Day 1 of the disease episode). On Day 2 POS he had a high fever (40.5° C). On Day 4 POS, suspecting bacterial otitis, he was prescribed amoxicillin. On Day 5 POS, he noticed a mild pain behind his eyes. On Day 9 POS, 1 day after defervescence, he noticed a slight rash on his trunk and extremities and interpreted this as allergy to the amoxicillin. 

Patient 2’s blood sample of 7 September (Day 37 POS) tested positive for IgM and IgG antibodies against DENV, with high titres (Table 2). The IgG response against JEV was interpreted as non-specific cross-reactivity. Also this patient had been vaccinated according to the Dutch national vaccination scheme. 

## Public health measures

On August 27, upon confirmation of the serological results, Patient 1 was reported by the RIVM to the French authorities through the Early Warning and Response System of the European Union as an autochthonous DENV infection probably acquired in France with cross-border implication. The French authorities contacted the patient and announced the case by press release on 8 September 2020 [1]. Santé publique France advised that also other family members with symptoms should be tested. That identified Patient 2.

The other seven Dutch family members visiting the holiday home between 13 and 31 July did not develop any disease symptoms and neither did the individuals with whom Patient 1 stayed during the first week of August in the other house nearby. None of the household members had recently travelled outside Europe. 

Notification to the French authorities led to a prompt local public health response including mosquito control around the holiday home (with deltametrine and *Bacillus thuringiensis israelensis* on a 150 m radius) and door-to-door investigations in order to identify other cases and raising awareness among local healthcare professionals and the public [1]. 

## Discussion

Here we describe two patients with dengue from the same family, who acquired the disease in Department Var, southern France. The signs and symptoms, as well as the plasmocytosis, of Patient 1 were typical for dengue [2]. The list of differential diagnoses was therefore very short and the high IgM and IgG titres for DENV were considered confirmative, even though definite confirmation would require demonstration of virus or serodiagnosis on paired samples [3]. PCR was not conducted to detect DENV in blood or urine because the chance for a positive test was considered low in this rather late stage of disease and it was not deemed necessary for confirmation of disease, clinical management, notification or public health measures.

Dengue is endemic in large parts of the world and a common illness among returning travellers from (sub)tropical regions [4]. Because of globalisation in travel and trade and under changing ecological conditions, the geographical distribution of the vector of DENV, *Ae. albopictus*, gradually expanded over the last decades and may continue to do so [5]. Imported infections can continue to cause autochthonous outbreaks. A lack of awareness and a long interval between the viraemic episode of the patient with imported dengue and the first registration in the public health system were identified as possible drivers of local outbreaks [6]. *Aedes albopictus* has been established in France since 2004 and currently, the mosquito species is endemic in large parts of the country including one area close to the Belgian border [7,8]. The presence of *Ae. albopictus* in multiple sites in Europe means that also other diseases can be transmitted. Upon introduction by returning viraemic travellers, European cases of CHIKV, DENV and Zika virus infection have been reported [9,10]. As recently as August 2020, five patients in Vicenza province, northern Italy, were confirmed to have a DENV infection 2 weeks after a household member infected with DENV returned from West Sumatra [11]. Autochthonous DENV infection was first reported in France in 2010 and has since been reported at an almost yearly basis [12,13]. In 2020, by the end of September, six other autochthonous DENV cases had been reported by French authorities, one in the department Hérault and five in the department Alpes-Maritime [1,14,15]. Our case signalled the first evidence of local DENV activity in Département Var in 2020. In the recent past, between 2010 and 2019, six cases of autochthonous transmission were confirmed in the Départment Var [12]. However, our cases do not seem to have any connection with the other autochthonous cases identified in southern France this year.

## Conclusion 

The cases reported here again illustrate that travel medicine can have a role as a sentinel for detection of silent circulation of infectious diseases [16]. Clinicians should be aware of the possibility of ‘tropical’ vector-borne diseases acquired by travellers within European areas where competent vectors are present, even when cases have not been reported (yet) by local authorities. Rapid notification by clinicians and communication between national authorities is essential to ensure timely local risk management and disease control.
