# Authors’ correction for Euro Surveill. 2020;25(39)

**DOI:** 10.2807/1560-7917.ES.2020.25.39.201008c

**Published:** 2020-10-08

**Authors:** Eurosurveillance editorial team

**Affiliations:** 1 European Centre for Disease Prevention and Control (ECDC), Stockholm, Sweden

In the article "Autochthonous dengue in two Dutch tourists visiting Département Var, southern France, July 2020" by Vermeulen et al, published on 1 October 2020, the authors had omitted laboratory results for antibodies against West Nile and Zika virus. On request of the authors, these data were added to the text and to Table 2 on 8 October 2020.

